# Supplementary material for: The relationship between mitochondrial DNA haplotype and its copy number on body weight and morphological traits of Wuliangshan black-bone chickens
Source: PeerJ. 2024 Dec 16;12:e17989. doi: 10.7717/peerj.17989 (PMC11657187; doi:10.7717/peerj.17989)
Supplement: Supplemental Information 4 [file peerj-12-17989-s004.docx]

**Item to check**

**Experimental design qPCR oligonucleotides**

Definition of experimental and control groups E line126

Number within each group E line126

Assay carried out by the core or investigator’s laboratory? D line153

Acknowledgment of authors’ contributions D line320

**Sample**

Description E line125

Volume/mass of sample processed D line139

Microdissection or macrodissection E none

Processing procedure E line140

If frozen, how and how quickly? E ling140

If fixed, with what and how quickly? E none

Sample storage conditions and duration (especially for FFPEb samples) E line140

**Nucleic acid extraction**

Procedure and/or instrumentation E line141

Name of kit and details of any modifications E line141

Source of additional reagents used D none

Details of DNase or RNase treatment E none

Contamination assessment (DNA or RNA) E line143

Nucleic acid quantification E line143

Instrument and method E line144

Purity (A260/A280) D line143

Yield D none

RNA integrity: method/instrument E none

RIN/RQI or Cq of 3 and 5 transcripts E line172

Electrophoresis traces D none

Inhibition testing (Cq dilutions, spike, or other) E none

**Reverse transcription**

Complete reaction conditions E none

Amount of RNA and reaction volume E none

Priming oligonucleotide (if using GSP) and concentration E none

Reverse transcriptase and concentration E none

Temperature and time E none

Manufacturer of reagents and catalogue numbers D none

Cqs with and without reverse transcription Dc none

Storage conditions of cDNA D none

**qPCR target information**

Gene symbol E line157, line159

Sequence accession number E line157, line160

Location of amplicon D none

Amplicon length E none

In silico specificity screen (BLAST, and so on) E none

Pseudogenes, retropseudogenes, or other homologs? D none

Sequence alignment D none

Secondary structure analysis of amplicon D none

Location of each primer by exon or intron (if applicable) E none

What splice variants are targeted? E none

**qPCR oligonucleotides**

Primer sequences E line157-160

RTPrimerDB identification number D none

Probe sequences Dd none

Location and identity of any modifications E none

Manufacturer of oligonucleotides D none

Purification method D none

**qPCR protocol**

Complete reaction conditions E line163-165

Reaction volume and amount of cDNA/DNA E line161

Primer, (probe), Mg2, and dNTP concentrations E line162

Polymerase identity and concentration E none

Buffer/kit identity and manufacturer E none

Exact chemical composition of the buffer D none

Additives (SYBR Green I, DMSO, and so forth) E none

Manufacturer of plates/tubes and catalog number D none

Complete thermocycling parameters E line163

Reaction setup (manual/robotic) D manual

Manufacturer of qPCR instrument E line164

**qPCR validation**

Evidence of optimization (from gradients) D none

Specificity (gel, sequence, melt, or digest) E none

For SYBR Green I, Cq of the NTC E none

Calibration curves with slope and y intercept E none

PCR efficiency calculated from slope E line166

CIs for PCR efficiency or SE D none

r^2^ of calibration curve E none

Linear dynamic range E none

Cq variation at LOD E none

CIs throughout range D none

Evidence for LOD E none

If multiplex, efficiency and LOD of each assay E none

**Data analysis**

qPCR analysis program (source, version) E line172

Method of Cq determination E none

Outlier identification and disposition E none

**Results for NTCs E**

Justification of number and choice of reference genes E none

Description of normalization method E line172

Number and concordance of biological replicates D none

Number and stage (reverse transcription or qPCR) of technical replicates E line172

Repeatability (intraassay variation) E none

Reproducibility (interassay variation, CV) D none

Power analysis D none

Statistical methods for results significance E line195

Software (source, version) E line192

Cq or raw data submission with RDML D none
